# 16S–23S rRNA Gene Intergenic Spacer Region Variability Helps Resolve Closely Related Sphingomonads

**DOI:** 10.3389/fmicb.2016.00149

**Published:** 2016-02-11

**Authors:** Sima Tokajian, Nahla Issa, Tamara Salloum, Joe Ibrahim, Maya Farah

**Affiliations:** Department of Natural Sciences, School of Arts and Sciences, Lebanese American UniversityByblos, Lebanon

**Keywords:** sphingomonad, 16S–23S intergenic spacer sequence, ITS-2, phylogeny

## Abstract

Sphingomonads comprise a physiologically versatile group many of which appear to be adapted to oligotrophic environments, but several also had features in their genomes indicative of host associations. In this study, the extent variability of the 16S–23S rDNA intergenic spacer (ITS) sequences of 14 ATCC reference sphingomonad strains and 23 isolates recovered from drinking water was investigated through PCR amplification and sequencing. Sequencing analysis of the 16S–23S rRNA gene ITS region revealed that the ITS sizes for all studied isolates varied between 415 and 849 bp, while their G+C content was 42.2–57.9 mol%. Five distinct ITS types were identified: ITS^none^ (without tRNA genes), ITS^Ala(TGC)^, ITS^Ala(TGC)+Ile(GAT)^, ITS^Ile(GAT)+Ala(TGC)^, and ITS ^Ile(GAT)+Pseudo^. All of the identified tRNA^Ala(TGC)^ molecules consisted of 73 bases, and all of the tRNA^Ile(GAT)^ molecules consisted of 74 bases. We also detected striking variability in the size of the ITS region among the various examined isolates. Highest variability was detected within the ITS-2. The importance of this study is that this is the first comparison of the 16S–23S rDNA ITS sequence similarities and tRNA genes from sphingomonads. Collectively the data obtained in this study revealed the heterogeneity and extent of variability within the ITS region compared to the 16S rRNA gene within closely related isolates. Sequence and length polymorphisms within the ITS region along with the ITS types (tRNA-containing or lacking and the type of tRNA) and ITS-2 size and sequence similarities allowed us to overcome the limitation we previously encountered in resolving closely related isolates based on the 16S rRNA gene sequence.

## Introduction

Sphingomonads are Gram-negative, chemoheterotrophic, non-spore forming, straight rods, strictly aerobic, and characterized by an outer membrane containing glycosphingolipids as cell envelope components, but lacking lipopolysaccharide (Yabuuchi et al., 1990; White et al., 1996). Colonies are yellow-pigmented or whitish brown (Takeuchi et al., 1993). Sphingomonads are found in diverse natural environments playing an important role in nutrient cycling, especially in oligotrophic environments (Aylward et al., 2013). Some have been detected in plant and animal-associated environments, are being connected to rising health-care associated infections (Aylward et al., 2013; Narciso-da-Rocha et al., 2014), and were recently linked to peritoneal dialysis-associated peritonitis (Mohan and Railey, 2015). Sphingomonads are able to survive the chlorination of tap water and have the ability to co-aggregate and form biofilms. Large numbers of phenotypically and phylogenetically similar strains belonging to this group have been isolated. As a result Takeuchi et al. (2001) examined the complete 16S rRNA gene sequences, fatty acid profiles and polyamine patterns of several strains of the genus *Sphingomonas* and related genera. Based on the phylogenetic analyses of the 16S rRNA gene sequences and on some chemotaxonomic and phenotypic differences, the genus *Sphingomonas* was divided into four clusters and three new genera were proposed. Today sphingomonads encompass eight genera: *Novosphingobium*, *Sphingobium*, *Sphingomonas*, *Sphingopyxis*, *Sphingosinicella*, *Sphingomicrobium*, *Sphingorhabdus*, and *Parasphingopyxis* (Stolz, 2013).

The use of 16S rRNA gene sequence informatics to study bacterial phylogeny and taxonomy has been the most common housekeeping marker used (Janda and Abbott, 2007). However, many investigators have encountered resolution problems at the genus and/or species level due to the high level of similarity in the 16S rRNA gene sequence (Goncalves and Rosato, 2002; Janda and Abbott, 2007). This prompted the search for a new phylogenetic marker such as the 16S-23S rDNA intergenic spacer (ITS). The genes coding for ribosomal RNAs in prokaryotes are arranged in an operon in the following order 5′-16S–23S-5S-3′ and are separated by two spacer regions known as the ITS (Condon et al., 1995). ITS is more variable than the adjacent 16S and 23S ribosomal genes, and may be a better target for efficient identification at the species level due to its variability within a genus (Garcia-Martinez et al., 1996; Khan et al., 2005). This variability is due partly to differences in the number and type of tRNA sequences found within the spacer (Fredrickson et al., 1995).

Organisms isolated from a drinking water distribution network and water storage tanks in Lebanon were previously defined to be mainly Gram-negative, pigmented α-Proteobacteria belonging to the family of *Sphingomonadaceae* (Tokajian et al., 2008). 16S rRNA gene sequencing, biochemical identification using the Biolog system and restriction digestion of the amplified ITS region did not yield reproducible results or enough variability to properly cluster and/or identify those isolates. In the present report the ITS sequences of 14 ATCC reference sphingomonad strains and 23 isolates representing those previously recovered from drinking water (Tokajian et al., 2008), was determined. These data were used to assess the extent variability of the ITS sequences, and to examine the potential of using this genetic marker to differentiate and delineate systematic relationships between isolates that usually: don’t fit within recognized biochemical profiles, don’t generate acceptable identification according to commercial systems, have too few sequences deposited in nucleotide databases and share high level of similarity in the 16S rRNA gene sequence.

## Materials and Methods

### Bacterial Strains

**FIGURE 1:**
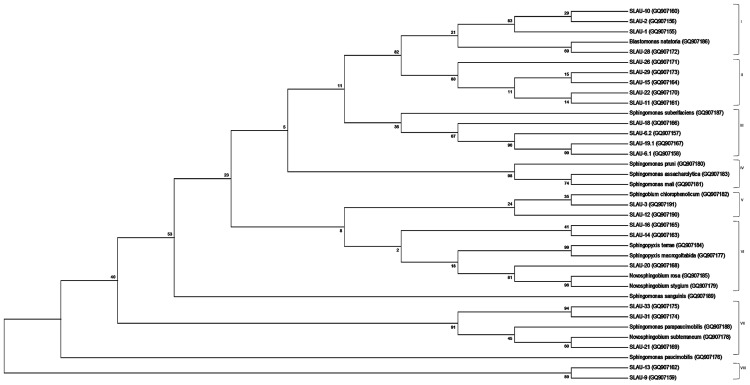
**Molecular Phylogenetic analysis by Maximum Likelihood method.** The evolutionary history was inferred by using the Maximum Likelihood method based on the Jukes-Cantor model. The bootstrap consensus tree inferred from 1000 replicates is taken to represent the evolutionary history of the taxa analyzed. The percentage of replicate trees in which the associated taxa clustered together in the bootstrap test (1000 replicates) are shown next to the branches. Initial tree(s) for the heuristic search were obtained by applying the Neighbor-Joining method to a matrix of pairwise distances estimated using the Maximum Composite Likelihood (MCL) approach. The analysis involved 37 nucleotide sequences of which 14 are references. All positions containing gaps and missing data were eliminated. There were a total of 86 positions in the final dataset. Evolutionary analyses were conducted in MEGA6.

The study was conducted using all forms and derivatives of yellow-pigmented colonies isolated from an intermittent drinking water distribution network (Tokajian et al., 2005) and Polyethylene and cast iron household storage tanks in Lebanon over a period of 2 years (Tokajian and Hashwa, 2004a,b). One hundred and twenty-nine Gram-negative rods with whitish to yellow-pigmented colonies were isolated and purified on R2A agar (Oxoid; Reasoner and Geldreich, 1985). The isolates were grouped into biotypes representing the various colony color and morphology obtained upon growing on R2A for 48 h at 28°C (**Figure 1**). Twenty-three isolates representing the different biotypes were chosen and designated as SLAU-(1–3) (GenBank accession numbers: GQ907155/56/91), SLAU-(6.1–6.2) (GenBank accession numbers: GQ907158/7), SLAU-(9–16) (GenBank accession numbers: GQ907159/60/61/90/62/63/64/65), SLAU-18 (GenBank accession number: GQ907166), SLAU-19.1 (GenBank accession number: GQ907167), SLAU-(20–22) (GenBank accession numbers: GQ907168/69/70), SLAU-26 (GenBank accession number: GQ907171), SLAU-(28–29) (GenBank accession numbers: GQ907172/73), SLAU-31 and SLAU-33 (GenBank accession numbers: GQ907174/75).

### Reference Strains

The following 14 ATCC reference strains were used: ATCC BAA-1092 *Sphingomonas paucimobilis*, ATCC 51380 *Sphingopyxis macrogoltabida*, ATCC 51382 *Sphingomonas sanguinis*, ATCC 700279 *Novosphingobium subterraneum*, ATCC 700280 *N. stygium*, ATCC 51838 *Sphingomonas pruni*, ATCC 51231 *Sphingomonas parapaucimobilis*, ATCC 51840 *Sphingomonas mali*, ATCC 53874 *Sphingobium chlorophenolicum*, ATCC 51839 *Sphingomonas asaccharolytica*, ATCC 51381 *Sphingopyxis terrae*, ATCC 51837 *N. rosa*, ATCC 49356 *Sphingomonas suberifaciens* and ATCC 35951 *Blastomonas natatoria*. Growth conditions (medium type, incubation time, and temperature) used were according to ATCC recommendations.

### DNA Extraction

DNA extraction was done using InstaGene matrix solution (BIO-RAD, München, Germany). Samples with low DNA concentration and/or quality, the extraction was repeated using QIAamp DNA Mini Kit (Qiagen, Hilden, Germany), and all according to the manufacturers’ instructions. Lysates were then stored at -20°C until further processing.

### Sphingomonad-Specific 16S rDNA-Based PCR Assay

The PCR mixture contained 2 μl DNA (200 ng/μl), 1 U of AmpliTaq Gold (Applied Biosystems, USA), 0.5 μM of the forward and reverse primers (**Table 1**), 0.2 mM of each deoxynucleoside triphosphate (dNTP), 2.5 mM MgCl_2_ and 1× PCR buffer in a final volume of 50 μl (Leys et al., 2004). The expected PCR amplicon was around 352 bp long and was visualized by ethidium bromide staining on 1.5% agarose gel using 1X TAE buffer. 16S rRNA gene amplification was used as a positive PCR control to ensure the integrity of the DNA (Tokajian et al., 2008).

**Table 1:** Primers, primer sequences, and cycling conditions used in the 16S rDNA and 16S–23S ITS PCR reactions.

Assay	Primer name	Primer Sequence (5′-3′)	Annealing temperature (*T*_a_)	Cycling Conditions
16S rDNA PCR	Sphingo 108f	GCGTAACGCGTGGGAATCTG	62° C	95° C→5 min; 50 cycles of 95°C→5 s,62°C→10 s,
	Sphingo 420r	TTACAACCCTAAGGCCTTC		74°C→30 s, and 74°C →2 min
16S–23S ITS PCR	16S–1511f	AAGTCGTAACAAGGTARCCG	60° C	95° C→12 min; 30 cycles of 94° C→30 s,
	23S–23r	YYGCCAAGGCATCCACC		60°C→30 s, 72°C→1 min, and 72°C→10 min
	1492f	AAGTCGTAACAAGGTAACC		
	115r	GGGTTBCCCCATTCRG		


### ITS DNA Amplification

For amplification of the 16S–23S ITS region, PCR was performed in a total volume of 20 μl using the primers 16S–1511f targeting the end of 16S rDNA, and the reverse primer 23S–23r targeting the beginning of the 23S rDNA^1^ or 1492f targeting the end of 16S rDNA and 115r targeting the 23S rDNA (**Table 1**) (Garcia-Martinez et al., 1999). PCR reactions contained 2 μl DNA (50 ng/μL), 200 μM dNTPs, 0.4 pmol of each primer, 1X PCR Buffer II (Applied Biosystems), 2.5 mM MgCl_2_, and 0.1 U of AmpliTaq Gold DNA polymerase (Applied Biosystems). The amplified products were then visualized by ethidium bromide staining on 1.5% agarose gel using 1X TAE buffer with reference PCR products used as positive controls. The PCR products were purified using ExoSAP-IT (USB Corp., Cleveland, OH, USA).

### DNA Sequencing Reaction

The amplicons were sequenced using the ABI Prism BigDye Terminator v3.1 Cycle Sequencing Kit (Applied Biosystems). Two sequencing reactions were performed for each sample. The sequencing reaction consisted of the BigDye premix, 0.2 pmol of either forward or reverse primer, and the cleaned PCR product in a total volume of 10 μl. The same primers used in the PCR were used for sequencing. All sequencing reactions were performed with 25 cycles of 96°C for 10 s, 50°C for 5 s, and 60°C for 4 min.

### Sequence Analysis and Phylogenetic Tree

Sequences obtained were analyzed on CLC Main Workbench v5.5 and deposited to GenBank under the accession numbers indicated above. Sequences were aligned using the Clustal Omega multiple sequence alignment program^2^ (Sievers et al., 2011) with default parameters. Phylogeny was inferred using the Maximum Likelihood method based on the Jukes–Cantor evolutionary model with the consensus tree inferred from 1,000 bootstrap replicates. The initial tree(s) for the heuristic search were obtained by applying the Neighbor-Joining method to a matrix of pairwise distances estimated using the Maximum Composite Likelihood (MCL) approach. All position containing gaps and missing data were eliminated, and the total data set was composed of 86 positions. Tree building along with visualization were done using the MEGA6 program^3^ (Tamura et al., 2013) (**Figure 1**).

## Results and Discussion

### Structure, ITS Sequences and Phylogenetic Analysis

Sphingomonads comprise a physiologically versatile group many of which appear to be adapted to oligotrophic environments, but several also had features in their genomes indicative of host associations (Aylward et al., 2013). Currently, little sequence data is available on the ITS region for sphingomonads. The ITS region has been increasingly used to differentiate bacterial species or strains which cannot be easily differentiated using the 16S rRNA gene (Man et al., 2010). This is the first comparison of the 16S–23S rDNA ITS sequence similarities and tRNA genes from sphingomonads, where we analyzed the phylogenetic relationships based on ITS sequencing for a number of chosen sphingomonad ATCC reference strains along with representative sphingomonads recovered from drinking water in Lebanon (Tokajian et al., 2008).

**FIGURE 2:**
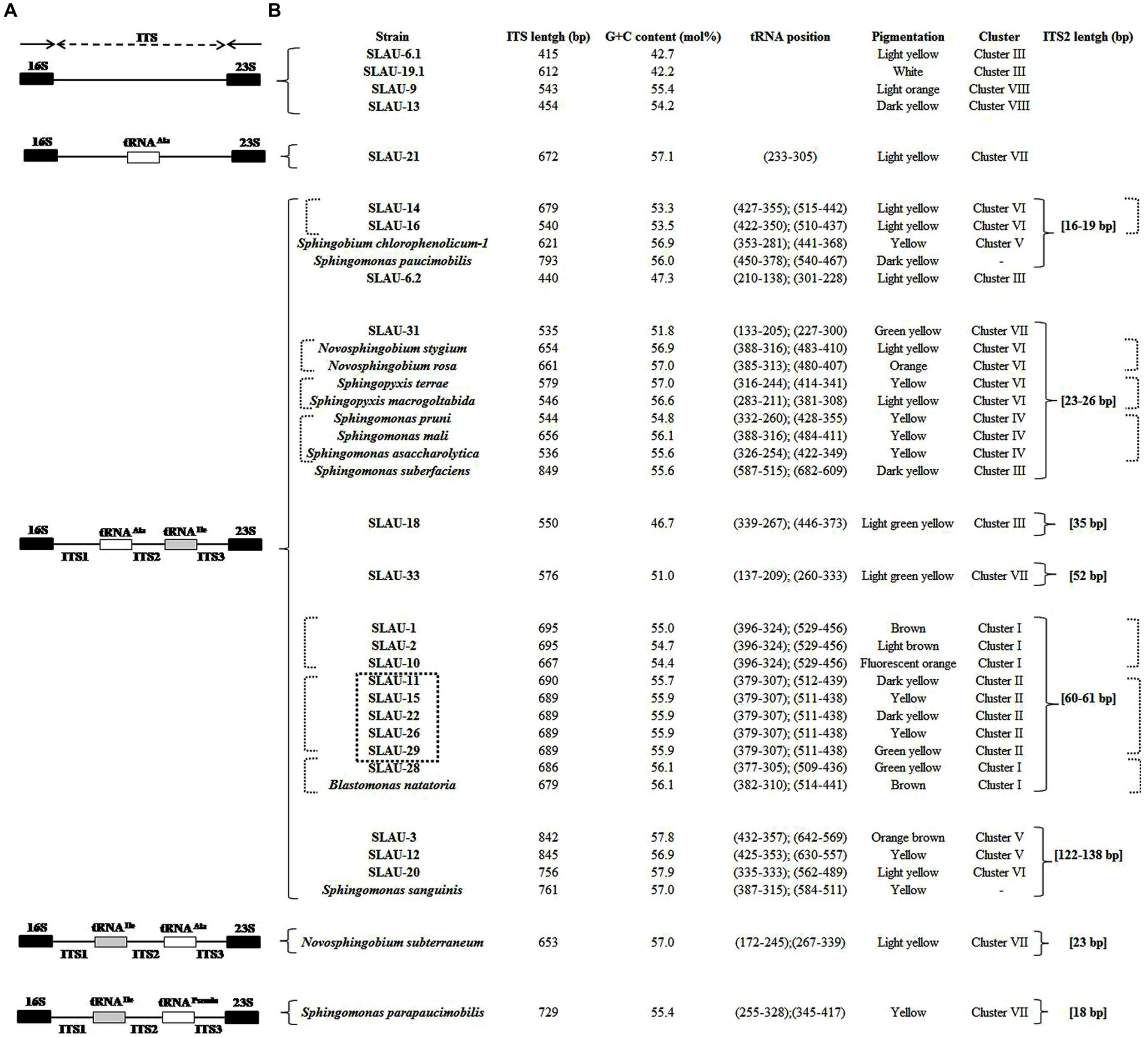
**(A)** Map showing different patterns observed in the 16S–23S rRNA ITS based on the tRNA (Ala and Ile) genes present. **(B)** The size of the different ITS was not respected. Table showing the length, G/C content, tRNA position, pigmentation, cluster based on the phylogenetic tree, and ITS-2 size of the isolates and reference strains included in this study. The position of each of the tRNAs is indicated in brackets, respectively. The dotted square indicates isolates having exactly identical ITS sequences. The dotted brackets indicate isolates having identical or similar ITS-2 sequences.

Previously Takeuchi et al. (2001) separated sphingomands into four clusters (Cluster I: *Sphingomonas*, Cluster II: *Sphingobium*, Cluster III: *Novosphingobium*, Cluster IV: *Sphingopyxis*) and considered each of the four clusters as a monophyletic and distinct phylogenetic group based on the 16S rDNA sequences. Our results however, revealed that discrepancies exist specially within Cluster I (*Sphingomonas* sp.). *S. parapaucimobilis* and *S. paucimobilis* formed distinct lines of descent (**Figure 1**). Since the ITS has a non-coding function, it is, therefore, susceptible to low selective pressure leading to extensive sequence mutation and insertion/deletion phenomena, making the ITS region more variable than the 16S rDNA (Tyrrell et al., 1997). ITS size of *S. parapaucimobilis* and *S. paucimobilis* was 729 and 793 bp, respectively. Similarly, *S. suberfaciens* (849 bp) clustered separately and had a larger ITS compared to *S. mali, S. pruni*, and *S. assacharolytica* (536–656 bp) (**Figures 1** and **2**).

**FIGURE 3:**
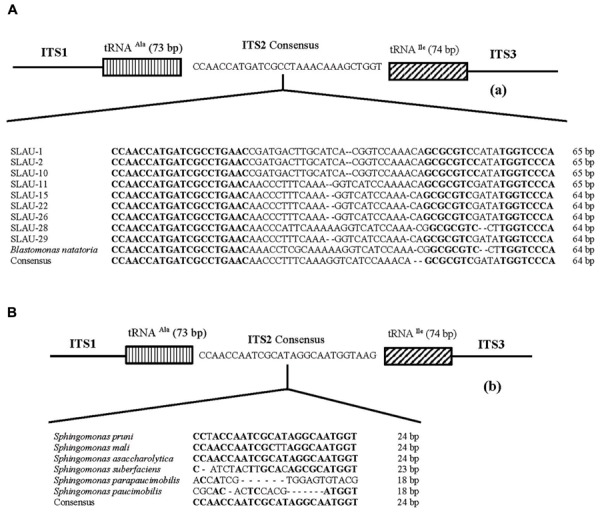
**Representative overall structure of the 16S–23S rDNA gene ITS region of the **(A)***Blastomonas natatoria* cluster and **(B)***Shingomonas* sp. showing the location of the tRNA genes and the nucleotide sequence of ITS-2.** Dashes represent single nucleotide deletions while conserved nucleotide blocks are indicated in bold; the consensus sequence is written below.

**FIGURE 4:**
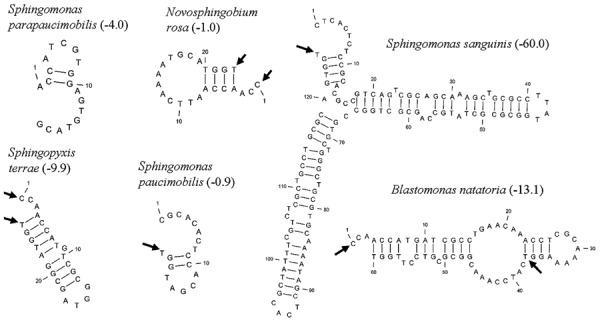
**Putative secondary structures of ITS2 representatives, arrows indicate the 5′ and 3′ ends of the 23 bp consensus sequence, while the values in parenthesis indicate free energy in kcal/mol**.

Five distinct ITS types were identified: ITS^none^ (without tRNA genes), ITS^Ala^ (with the tRNA^Ala^ gene), ITS^Ala(TGC)+Ile(GAT)^ (with tRNA^Ala^ and tRNA^Ile^ genes), ITS^Ile(GAT)+Ala(TGC)^ (with tRNA^Ile^ and tRNA^Ala^ genes) and ITS ^Ile(GAT)+Pseudo^ (with tRNA^Ile^ and tRNA^Pseudo^ genes) (**Figure 2A**). All of the identified tRNA^Ala(TGC)^ molecules consisted of 73 bases, and all of the tRNA^Ile(GAT)^ molecules consisted of 74 bases. ITS^none^ is rarely found in Gram-negative bacteria and was previously detected only in *Klebsiella* sp. (Wang et al., 2008) and some Gram-positive bacteria including *Staphylococcus aureus, Listeria monocytogenes*, and *Bacillus cereus* (Boyer et al., 2001). We also detected striking variability in the size of the ITS region among the various examined isolates (**Figure 2B**). Even within isolates showing the same pattern, the sizes of the individual ITS region was often different. The most common pattern was ITS^Ala+Ile^, which was detected in all of the studied ATCC reference strains except *S. parapaucimobilis*. The size range of this group was 440–849 bp, and in all except for *N. subterraneum*, the tRNA^Ala^ is just downstream of the 16S rRNA gene and the tRNA^Ile^ is just upstream of the 23S rRNA (**Figure 2B**). This was contrary to what has been previously reported, with the common arrangement being ITS^Ile+Ala^ (Boyer et al., 2001). However, *Xylella fastidosa* and *Campylobacter* sp. were also among the isolates reported to have the ITS^Ala+Ile^ arrangement (Simpson et al., 2000; Man et al., 2010).These tRNAs divided the ITS sequence into three parts: ITS-1, ITS-2, and ITS-3. Positions and structures of tRNAs and the start and end of the ITS-2 regions were determined using tRNAscan-SE Search Server^4^ (Lowe and Eddy, 1997) (**Figure 2B**). Highest variability was detected within the ITS-2, where the percent sequence conservation ranged from 6 to 96% (mean 22.5% ± 20.4%). This remarkable variation was also previously observed within the ITS-2 of *Xanthomonas* species (Goncalves and Rosato, 2002). Differences in the size of ITS-2 was also detected, dividing the isolates into different groups; the shortest having 16–19 nt and the longest 122–138 nt (**Figure 2B**). These groups were polyphyletic and did not correlate with the distribution of the isolates in the phylogenetic tree (**Figures 1** and **2B**), except for those having ITS-2 size of 60–61 nt and which included *Blastomonas natatoria* as the only ATCC reference strain. However, in this group there was a remarkable consensus within the whole ITS and ITS-2 regions with minor differences; a 10 bp deletion in the ITS region detected in *B. natatoria* at position 672–681 and only three insertion/deletion instances within ITS-2 (**Figure 3A**). This was in perfect harmony with the fact that *B. natatoria* not only represented a different line of descent from all other sphingomonads, but also differed in having photosynthetic and phytopathogenic traits (Takeuchi et al., 2001). Additionally, and in line with our previous observation, the highest ITS-2 sequence conservation within cluster I (*Sphingomonas* sp.) was detected in *S. mali, S. pruni*, and *S. assacharolyitca* (**Figure 3B**), which had the exact same size and almost identical sequences except for one base substitution in *S. mali* and *S. pruni*. Moreover, *N. stygium* and *N. rosa* had the same ITS-2 size and sequence, which differed slightly from that of *N. subteraneuem*. These findings revealed the heterogeneity and extent of variability within the ITS region as compared to the 16S rRNA gene even within closely related isolates. On the other hand, four of the sequenced isolates including the reference strain *S. sanguinis* exhibited longer sequences, 122–138 bp, which is suggestive of a common origin (**Figure 2B**). A longer stem was observed which provided more stability (**Figure 4**). The ones with shorter sequences 16–19 bp, formation of a secondary hairpin structure could be predicted, which could represent a putative target for RNase III during the processing of tRNA^ala^ and tRNA^Ile^ (**Figure 4**). The longer observed sequences are either due to the addition or deletion of three long stretches of nucleotides at different positions (positions: 23–43, 43–94, and 95–137) with a size of 20–51 nt. Finally, it is noteworthy that all the reference strains had a conserved nucleotide block of TGGT (except *S. parapaucimobilis* it was TACG and in *N. subterraneum* it was TTGG) at the end of the ITS-2 and some consensus sequences such as CCAACCAT at the beginning.

Sequencing the ITS region and examining the variability within the ITS-2 helped in overcoming limitations we previously encountered using 16S rRNA gene sequences and whether with the ATCC reference strains or the unknown isolates we were able to better understand the phylogeny of those isolates. The availability of few 16S rRNA gene sequences deposited in nucleotide databases and the similarities that existed in the 16S rRNA sequences led to poor discriminatory power (Tokajian et al., 2008). Although with some of the sequenced isolates a definitive identification was not attained using the ITS sequencing approach, but absolute resolution was clearly observed. Moreover, this study revealed discrepancies in Cluster I (*Sphingomonas* sp.), which calls for careful reconsideration.

### The G+C Content of ITS Sequences

The total G+C content in sphingomonads was 62-68% (Takeuchi et al., 2001), while in the ITS sequence the range was 42.2–57.9 mol%. This was in line with what was previously observed in *Xanthomonas* species*, Salmonella typhimurium* and *Escherichia coli*, which confirms that the selective pressure is not identical in the coding and non-coding regions (Syvanen, 1994; Goncalves and Rosato, 2002).

## Conclusion

Although 16S rRNA gene sequencing has been widely used for typing bacterial isolates, it was shown previously that this region does not provide enough information to discern between closely related bacterial strains at the sub-generic level, especially for diverging species (Fox et al., 1992). Sequence and length polymorphisms of ITS regions have been increasingly used as tools for the identification of bacterial species and/or subspecies, where the ITS region is hypervariable when compared to the more conserved 16S rDNA (Garcia-Martinez et al., 1999), and hence sequencing of the 16S–23S ITS region provided more information for the identification at the species and the subspecies levels (Gurtler and Stanisich, 1996; Ernst et al., 2003; Xu and Cote, 2003). Collectively the data obtained in this study show that sequence and length polymorphisms within the ITS region along with the ITS types (tRNA-containing or lacking and the type of tRNA) and ITS-2 size and sequence similarities can all be used as a potentially powerful tool to study the phylogeny of such isolates and to delineate systematic relationships. Additionally, and based on ITS sequencing some of the unidentifiable drinking water isolates, which were phenotypically similar to sphingomonads and were not identified using the 16S rRNA gene sequencing (Tokajian et al., 2008), had ITS sequences with sufficient variations that allowed to overcome the limitation of resolving closely related isolates based on the 16S rRNA gene sequence. Moreover, the ITS sequence informatics could help clinical settings in resolving the problem of identifying organisms that are rarely associated with human infections and that usually don’t fit within recognized biochemical profiles.

It is noteworthy, the high-throughput genome sequencing of a number of sphingomonads revealed the presence of a large number of species-specific genes with few genomic features that can reliably be used to differentiate between the genera. These observed discrepancies were attributed to the presence of selfish genetic elements playing a significant role in shaping genome evolution, along with megaplasmids, transposons, plasmids, and chromosomal rearrangements (Aylward et al., 2013). Due to selective pressures few genomic features within sphingomonads can reliably distinguish between the different genera with organisms belonging to the different clusters exhibiting a high genomic plasticity (Aylward et al., 2013; Narciso-da-Rocha et al., 2014). Finally, in light of the findings of this study, and as the 16S rRNA gene sequencing alone cannot be used as a reliable genetic marker for sphingomonads, high-throughput genome sequencing of isolates representing the four clusters proposed by Takeuchi et al. (2001) is highly recommended.

## Author Contributions

Conceived and designed the experiments: ST and MF; performed the experiments: NI and MF; analyzed the data: ST, MF, TS, and JI; and wrote the paper: ST.

## Conflict of Interest Statement

The authors declare that the research was conducted in the absence of any commercial or financial relationships that could be construed as a potential conflict of interest.

## Footnotes

1 http://www.ridom.de/rdna/

2 http://www.clustal.org/omega/

3 http://www.megasoftware.net/

4 lowelab.ucsc.edu/tRNAscan-SE/
